# Pan-Cancer Analysis of the Prognostic and Immunological Role of HSF1: A Potential Target for Survival and Immunotherapy

**DOI:** 10.1155/2021/5551036

**Published:** 2021-06-18

**Authors:** Fei Chen, Yumei Fan, Pengxiu Cao, Bing Liu, Jiajie Hou, Bo Zhang, Ke Tan

**Affiliations:** Key Laboratory of Animal Physiology, Biochemistry and Molecular Biology of Hebei Province, Ministry of Education Key Laboratory of Molecular and Cellular Biology, College of Life Sciences, Hebei Normal University, Shijiazhuang, Hebei 050024, China

## Abstract

Emerging evidence revealed the significant roles of heat shock factor 1 (HSF1) in cancer initiation, development, and progression, but there is no pan-cancer analysis of HSF1. The present study first comprehensively investigated the expression profiles and prognostic significance of HSF1 and the relationship of HSF1 with clinicopathological parameters and immune cell infiltration using bioinformatic techniques. HSF1 is significantly upregulated in various common cancers, and it is associated with prognosis. Pan-cancer Cox regression analysis indicated that the high expression of HSF1 was associated with poor overall survival (OS), disease-specific survival (DSS), and progression-free interval (PFI) in cervical squamous cell carcinoma and endocervical adenocarcinoma (CESC), head and neck squamous cell carcinoma (HNSC), and kidney renal papillary cell carcinoma (KIRP) patients. The methylation of HSF1 DNA was decreased in most cancers and negatively correlated with the HSF1 expression. Increased phosphorylation of S303, S307, and S363 in HSF1 was observed in some cancers. HSF1 remarkably correlated with the levels of infiltrating cells and immune checkpoint genes. Our pan-cancer analysis provides a deep understanding of the functions of HSF1 in oncogenesis and metastasis in different cancers.

## 1. Introduction

Cancer is a leading cause of morbidity and mortality worldwide, and it imposes a major health and economic burden on society [1]. Unfortunately, the number of newly diagnosed cases continues to increase, and the burden of cancer will undoubtedly worsen. Current, cancer treatment strategies primarily include surgery, chemotherapy, radiotherapy, targeted therapy, and immunotherapy [1]. Although these therapies exhibit some clinical success, the prognosis and survival rate of patients remain unsatisfactory due to drug resistance, side effects, and other problems [2]. Therefore, it is urgent to actively search for other therapeutic targets and novel sensitive tumor biomarkers for the diagnosis and treatment of cancer [3].

Oncogenesis is a multistep, multilayered process that includes oncogene activation, inhibition of tumor suppressor genes, genomic instability, epigenetic alteration modifications, and abnormal cell signaling, which lead to the production of abnormal proteins and stress signals [4, 5]. The lack of nutrients and oxygen, ATP depletion, and the inflammatory response in the tumor microenvironment (TME) creates a long-term stressful living environment for tumor cells [6, 7]. This stress signaling leads to the activation of heat shock factor 1 (HSF1), which induces unique transcriptional programs to address these alterations [8–10]. Consistent with these observations, a growing number of studies showed that HSF1 was overexpressed and/or activated in various types of cancer and negatively associated with the prognosis of cancer patients [8–10].

HSF1 is an evolutionarily conserved master regulator of the heat shock response (HSR), which mediates the expression of downstream heat shock proteins (HSPs) at the transcriptional level to support cellular protein homeostasis by facilitating nascent protein synthesis, protein folding, and protein degradation [11–13]. Heat shock is a typical stimulus for HSF1 activation, but it is also activated by other stresses, such as heavy metals, radiation, and oxidative stress [14, 15]. Recent research showed that HSF1 promoted tumorigenesis via a variety of ways, including maintaining proteostasis, reprogramming metabolism, facilitating cancer cell proliferation and migration, repairing the genome, preventing cell death, and altering the TME [8, 16, 17]. Therefore, the targets of HSF1 include HSPs and many other oncogenesis-promoting genes [18, 19]. Recent studies also demonstrated that HSF1-regulated target genes in tumor cells did not overlap with the target genes regulated by heat stress, which suggests a special regulatory role of HSF1 in tumor development and progression [18]. However, most studies on the function of HSF1 in cancers were limited to a specific type of cancer. Therefore, it is particularly important to deeply examine the regulatory functions and molecular mechanisms of HSF1 in a pan-cancer dataset to provide new directions and strategies for the clinical treatment of cancer.

The present study systematically characterized the prevalence and prognostic value of HSF1 expression in pan-cancer. We combined data from different databases, including The Cancer Genome Atlas (TCGA), UALCAN, Kaplan-Meier Plotter, TIMER, and cBioPortal, to investigate the roles of HSF1 in prognosis and the immune response. We evaluated the potential correlations between the HSF1 expression and tumor mutational burden (TMB), microsatellite instability (MSI), DNA methylation, immune infiltration levels, and various immune-related genes across multiple cancer types. We also examined the biological function and pathways of HSF1 using the Kyoto Encyclopedia of Genes and Genomes (KEGG) and Gene Set Enrichment Analysis (GSEA). We found that the HSF1 expression was abnormally upregulated and negatively correlated with DNA methylation. The high expression of HSF1 significantly correlated with a poor prognosis of several types of cancer. Remarkably, the phosphorylation of the S303, S307, and S363 residues on HSF1 was increased in some cancers. HSF1 upregulation was associated with the increased infiltration of immune cells, including B cells, CD4+ T cells, CD8+ T cells, neutrophils, macrophages, and dendritic cells (DCs). The HSF1 expression exhibited strong correlations with immune checkpoint genes according to pan-cancer analysis. Our pan-cancer analysis provides a deep understanding of the functions of HSF1 in oncogenesis in different cancers and identifies strategies that may be used to promote collaborative activities in the context of immunotherapy.

## 2. Materials and Methods

### 2.1. Tumor Immune Estimation Resource (TIMER)

The HSF1 expression profile and the abundance of immune infiltrates in pan-cancer were analyzed using the TIMER database (https://cistrome.shinyapps.io/timer/). The gene expression levels are represented as log2 TPM values.

### 2.2. HSF1 Expression Pattern in Human Pan-Cancer

The dysregulation of the HSF1 expression between various types of cancer and normal tissues was investigated by combining the data for normal tissues from the GTEx database with data from The Cancer Genome Atlas (TCGA). RNA sequencing data and clinical follow-up information for patients with 33 types of cancers, including adrenocortical carcinoma (ACC), bladder urothelial carcinoma (BLCA), breast invasive carcinoma (BRCA), cervical squamous cell carcinoma (CESC), cholangiocarcinoma (CHOL), colon adenocarcinoma (COAD), lymphoid neoplasm diffuse large B cell lymphoma (DLBC), esophageal carcinoma (ESCA), glioblastoma (GBM), brain lower grade glioma (LGG), head and neck squamous cell carcinoma (HNSC), kidney chromophobe (KICH), kidney renal clear cell carcinoma (KIRC), kidney renal papillary cell carcinoma (KIRP), acute myeloid leukemia (LAML), liver hepatocellular carcinoma (LIHC), lung adenocarcinoma (LUAD), lung squamous cell carcinoma (LUSC), mesothelioma (MESO), ovarian serous cystadenocarcinoma (OV), pancreatic adenocarcinoma (PAAD), pheochromocytoma and paraganglioma (PCPG), prostate adenocarcinoma (PRAD), rectum adenocarcinoma (READ), sarcoma (SARC), skin cutaneous melanoma (SKCM), stomach adenocarcinoma (STAD), testicular germ cell tumors (TGCT), thyroid carcinoma (THCA), thymoma (THYM), uterine corpus endometrial carcinoma (UCEC), uterine carcinosarcoma (UCS), and uveal melanoma (UVM), were obtained from the TCGA database. All expression data were normalized via log2 conversion.

### 2.3. Prognostic Analysis

The connection between the HSF1 expression and the prognosis of patients, including overall survival (OS), disease-specific survival (DSS), disease-free interval (DFI), and progression-free interval (PFI) in 33 types of cancer was examined using forest plots and Kaplan-Meier curves. The hazard ratios (HRs) and 95% confidence intervals were calculated using univariate survival analysis.

### 2.4. UALCAN

The UALCAN database (http://ualcan.path.uab.edu/analysis.html) was used to investigate the methylation level and phosphorylation of HSF1 between different cancers and corresponding adjacent tissues. The significance of differences was evaluated using Student's *t*-test, and *p* < 0.05 was considered statistically significant.

### 2.5. KEGG and GSEA

KEGG analyses were used to examine the biological and molecular functions of HSF1 in COAD. We also used GSEA to determine the potential molecular mechanisms of HSF1 in COAD. KEGG and GSEA were performed using the *R* package ClusterProfiler.

### 2.6. Pan-Cancer Analysis of the Correlation of the HSF1 Expression with Tumor Cell Infiltration and Immune Modulator Genes

The data of 33 types of cancer and normal tissues in TCGA were downloaded from the Genomic Data Commons (GDC) data portal website. For reliable immune score evaluation, we used Immuneeconv, which is an *R* software package that integrates the two latest algorithms, TIMER and xCell. A Spearman correlation analysis heat map of the immune score or immune checkpoint-related genes and HSF1 gene expression in multiple cancers was generated. The horizontal axis in heat maps represents different types of cancer, the vertical axis represents different immune scores, and different colors represent correlation coefficients. *R* software v4.0.3 was used for statistical analysis (^∗^*p* < 0.05, ^∗∗^*p* < 0.01, ^∗∗∗^*p* < 0.001).

### 2.7. Pan-Cancer Analysis of the Relationship between the HSF1 Gene Expression and TMB or MSI

The TMB and MSI scores were obtained from TCGA. Correlation analysis between the HSF1 expression and TMB or MSI was performed using Spearman's method. The horizontal axis in the figure represents the correlation coefficient between HSF1 and TMB or MSI, the ordinate is different types of cancer, the size of the dots in the figure represents the size of the correlation coefficient, and the different colors represent the significance of the *p* value.

### 2.8. Statistical Analysis

Alterations in HSF1 expression levels in cancer tissues and normal tissues were estimated using *t*-tests. For survival analysis, the HR and *p* value were calculated using univariate Cox regression analysis. Kaplan-Meier analysis was used to investigate the survival time of patients stratified according to high or low levels of the HSF1 expression. *p* < 0.05 was set as the significance threshold for all statistical analyses.

## 3. Results

### 3.1. Pan-Cancer Expression Landscape of HSF1

**Figure 1:**
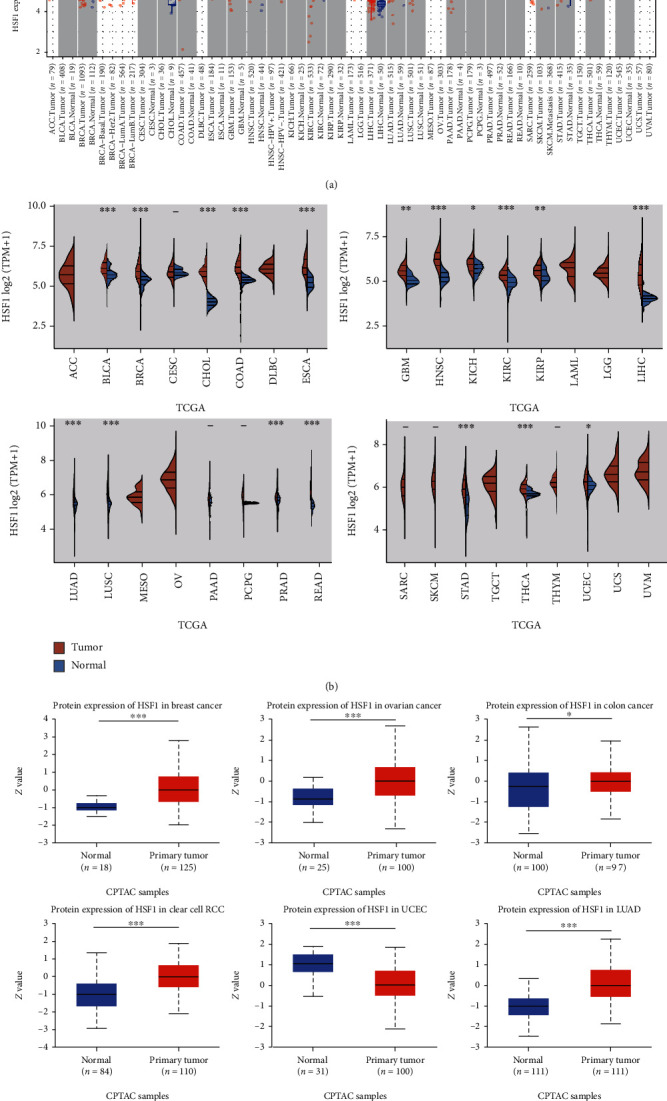
Upregulated mRNA expression of HSF1 in pan-cancer. (a) The results from the TIMER database indicated that the HSF1 expression was remarkably increased in 16 cancer types. The red and blue boxes represent tumor tissues and normal tissues, respectively. (b) The expression level of HSF1 in different cancer types from TCGA. (c) The HSF1 protein expression level in normal tissues and primary tissues of breast cancer, ovarian cancer, colon cancer, clear cell RCC, and UCEC was examined using the CPTAC dataset. ^∗^*p* < 0.05, ^∗∗^*p* < 0.01, and ^∗∗∗^*p* < 0.001.

According to the results from the TIMER database, HSF1 exhibited inconsistent mRNA expression in 34 types of human common cancer. The HSF1 expression was significantly higher in cancer versus adjacent normal tissues in the BLCA, BRCA, CHOL, COAD, ESCA, HNSC, KICH, KIRC, KIRP, LIHC, LUAD, LUSC, PRAD, READ, STAD, and THCA datasets (Figure 1(a)). We also compared the HSF1 expression using the data directly from the TCGA. The upregulated HSF1 mRNA expression was observed consistently in tumor tissues versus normal tissues in the BLCA, BRCA, CHOL, COAD, ESCA, GBM, HNSC, KICH, KIRC, KIRP, LIHC, LUAD, LUSC, PRAD, READ, STAD, THCA, and UCEC datasets (Figure 1(b)).

Further comparison of the HSF1 protein expression according to the Clinical Proteomic Tumor Analysis Consortium (CPTAC) database demonstrated that the HSF1 protein expression was significantly increased in advanced tumor tissues versus normal tissues in breast cancer, ovarian cancer, colon cancer, clear renal cell carcinoma (RCC), and LUAD, but it was decreased in UCEC (Figure 1(c)).

### 3.2. Pan-Cancer Analysis of the Correlation between HSF1 Expression and Clinicopathology

**Figure 2:**
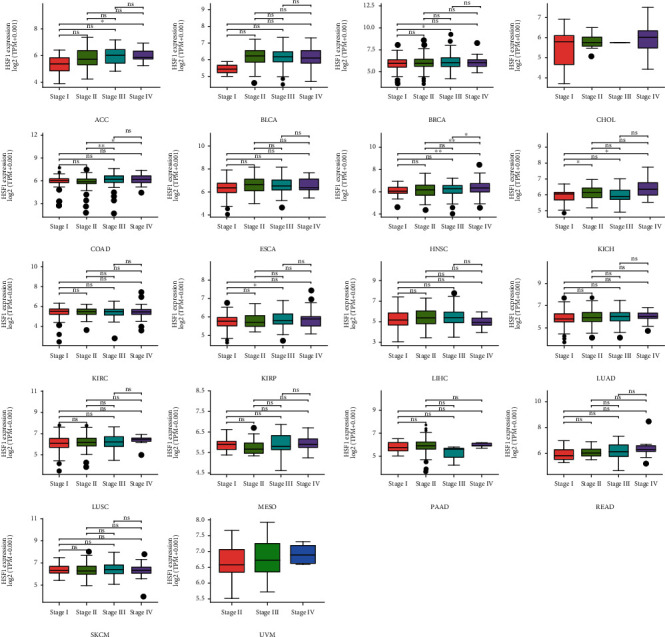
Correlations between the HSF1 expression and the main pathological stages, including stage I, stage II, stage III, and stage IV of ACC, BLCA, BRCA, CHOL, COAD, ESCA, HNSC, KICH, KIRC, KIRP, LIHC, LUAD, LUSC, MESO, PAAD, READ, SKCM and UVM, were investigated based on the TCGA data. Log2 (TPM+1) was used for log scale. ^∗^*p* < 0.05, ^∗∗^*p* < 0.01, and ^∗∗∗^*p* < 0.001.

To investigate the association between the HSF1 expression and clinicopathological features in multiple cancers, we assessed the HSF1 expression in stage I, II, III, and IV, cancer patients. The results from the TCGA database revealed that the expression of HSF1 was significantly upregulated in ACC, BRCA, COAD, HNSC, KICH, and KIRP (Figure 2). The HSF1 expression was consistent in several advanced cancers, including BLCA, ESCA, KIRC, LIHC, LUAD, LUSC, MESO, PAAD, READ, and SKCM (Figure 2).

### 3.3. Pan-Cancer Analysis of the Multifaceted Prognostic Value of HSF1

**Figure 3:**
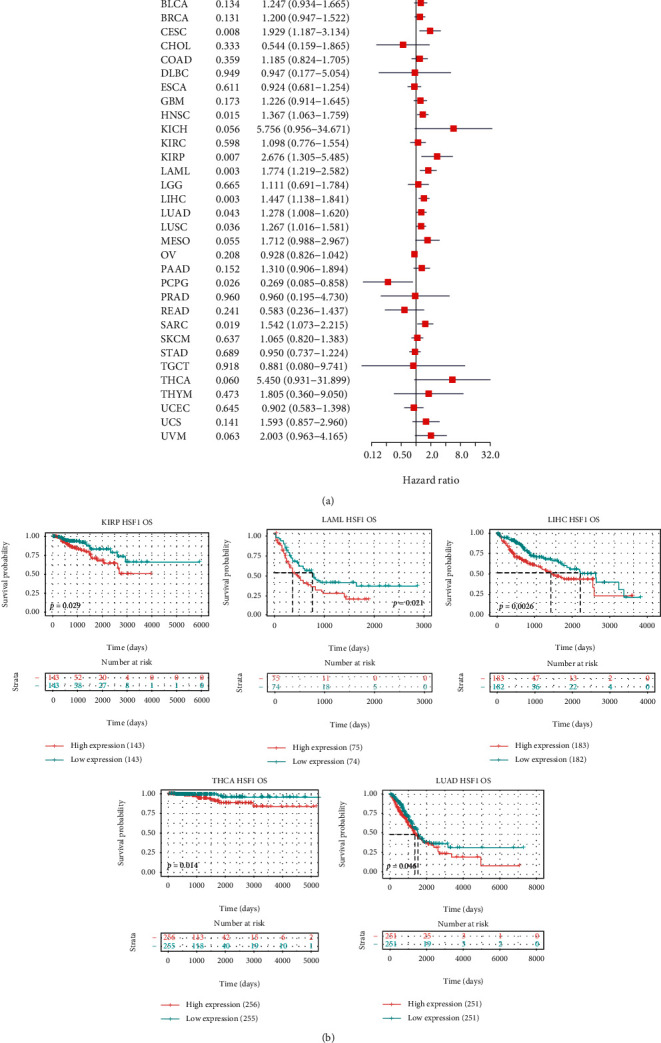
Association between the HSF1 expression and the OS of cancer patients. (a) A forest plot of hazard ratios of HSF1 in 33 types of tumors. (b) Kaplan-Meier survival curves of OS for patients stratified by the different expressions of HSF1 in LAML, LUAD, LIHC, KIRP, and THCA.

**Figure 4:**
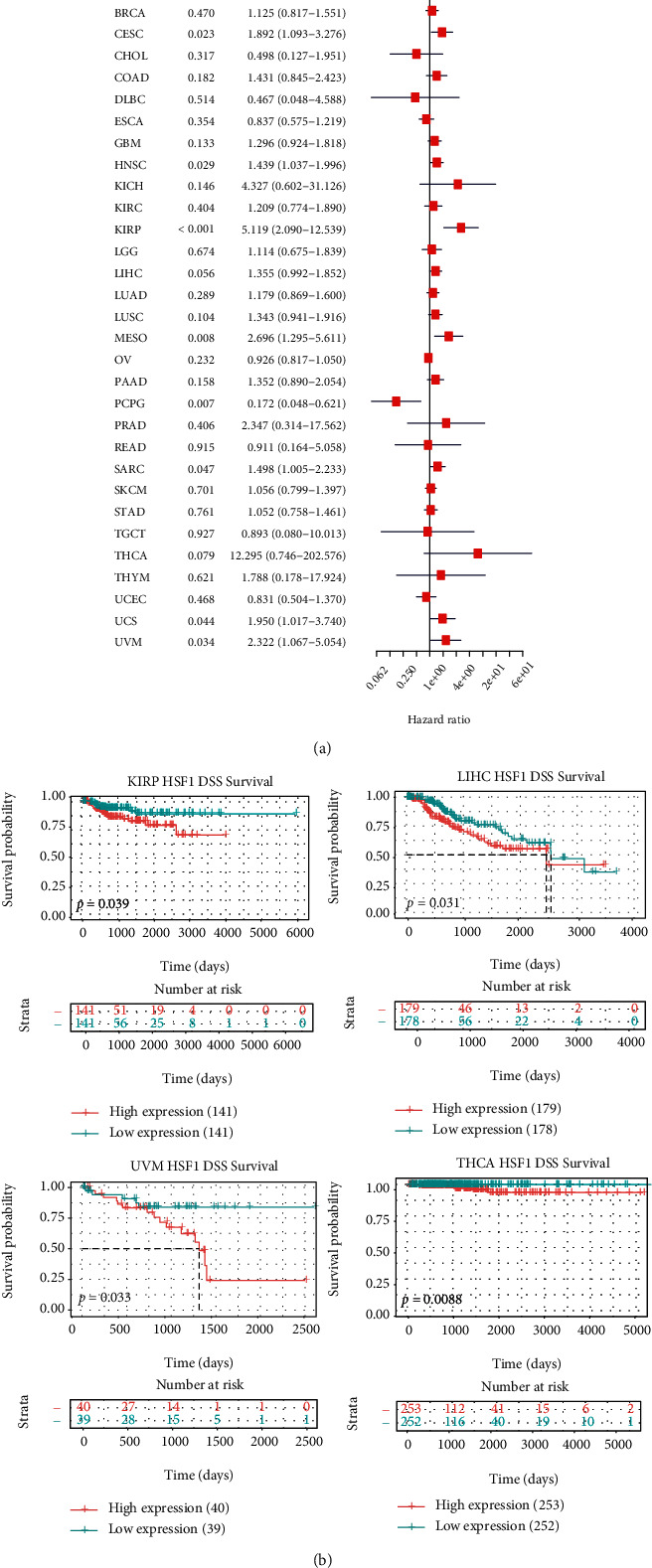
Association between the HSF1 expression and DSS in cancer patients. (a) A forest plot of hazard ratios of HSF1 in 33 types of tumors. (b) Kaplan-Meier survival curves of DSS for patients stratified by the different expressions of HSF1 in LIHC, KIRP, THCA, and UVM.

We estimated the association between the HSF1 expression and the prognosis of patients in the pan-cancer dataset. The survival metrics included OS, DSS, DFI, and PFI. Cox regression analysis of the results from 33 types of cancer suggested that the HSF1 expression significantly correlated with OS in 10 types of cancer, including ACC, CESC, HNSC, KIRP, LAML, LIHC, LUAD, LUSC, PCPG, and SARC (Figure 3(a)). Kaplan–Meier survival curves indicated that the upregulated HSF1 expression was remarkably associated with poor OS in LAML, LIHC, LUAD, KIRP, and THCA (Figure 3(b)). We examined the relationship between the HSF1 expression and DSS in cancer patients. The HSF1 expression affected DSS in eight types of cancer, including CESC, HNSC, KIRP, MESO, PCPG, SARC, USC, and UVM (Figure 4(a)). Kaplan–Meier analysis indicated that the increased HSF1 expression corresponded with poor DSS in patients with KIRP, LIHC, THCA, and UVM (Figure 4(b)). Cox regression analysis of the PFI demonstrated that the increased HSF1 expression was a risk factor in ACC, CESC, HNSC, KICH, KIRP, LUSC, PCPG, PRAD, and UVM (Supplementary Figure 1(a)). The results from Kaplan–Meier analysis suggested that the increased HSF1 expression was associated with a poor prognosis in four types of cancer, namely, ACC, HNSC, PRAD, and UCS (Supplementary Figure 1(b)). We also assessed the association between the HSF1 expression and DFI and identified that the HSF1 expression influenced DFI in patients with ACC, COAD, and PRAD (Supplementary Figure 2(a)). Kaplan-Meier DFI curves revealed that the increased HSF1 mRNA expression correlated with unfavorable DFI in ACC and TGCT (Supplementary Figure 2(b)).

### 3.4. Pan-Cancer Analysis of the Methylation Level and Genetic Alteration of HSF1

**Figure 5:**
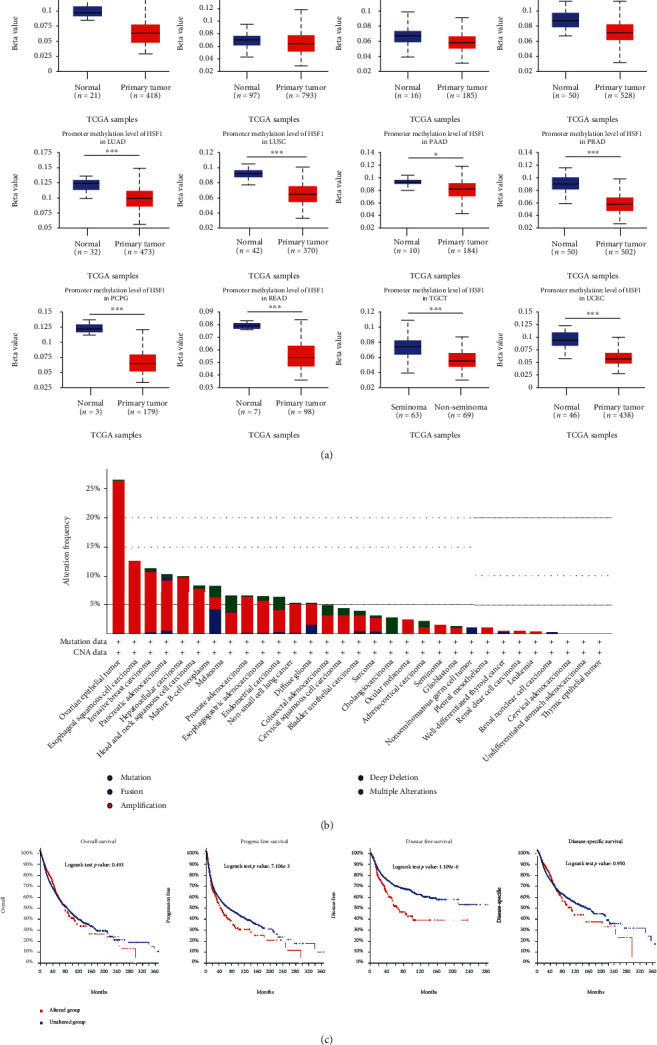
DNA methylation and mutation features of HSF1 in pan-cancer. (a) Promoter methylation level of HSF1 in pan-cancer. The results were obtained from the UALCAN database. (b) The alteration frequency with different types of mutations was examined using the cBioPortal database. (c) The effect of HSF1 mutation status on overall, disease-specific, disease-free, and progression-free survival of cancer patients was investigated using the cBioPortal database. ^∗^*p* < 0.05, ^∗∗^*p* < 0.01, and ^∗∗∗^*p* < 0.001.

DNA methylation directly affects cancer occurrence and progression [20]. We investigated the DNA methylation of HSF1 using the UALCAN and TCGA databases. A significant decrease in the methylation level of HSF1 was observed in BLCA, BRCA, ESCA, HNSC, LIHC, LUAD, LUSC, PAAD, PRAD, PCPG, READ, TGCT, and UCEC tissues compared to normal tissues according to the UALCAN database (Figure 5(a)). The methylation level of HSF1 in KIRC and KIRP was greatly increased (Supplementary Figure 3). However, no differences were observed between CHOL, COAD, CESC, GBM, SARC, STAD, THCA, and THYM tissues and matched normal tissues (Supplementary Figure 3). Data from the TCGA database revealed that the DNA methylation levels of HSF1 negatively correlated with the HSF1 expression in ACC, BLCA, BRCA, CESC, CHOL, COAD, ESCA, HNSC, LGG, LIHC, LUAD, LUSC, MESO, OV, PAAD, PCPG, PRAD, READ, SARC, SKCM, STAD, TGCT, THYM, UCS, and UVM (Supplementary Figure 4).

We also investigated the pan-cancer alterations of HSF1 using the cBioPortal (TCGA, Pan-Cancer Atlas) database. The results demonstrated that the highest alteration frequency of HSF1 was approximately 27% in patients with ovarian epithelial tumors (Figure 5(b)). Among the different types of genetic alterations, amplification was the most common type. We also examined the potential relationship between genetic alterations in HSF1 and the prognosis of patients with different types of cancer. As shown in Figure 5(c), tumor patients with genetic alterations in HSF1 had worse progression-free survival (PFS) and disease-free survival (DFS) than patients without alterations, but OS and DSS were not different between the two groups.

### 3.5. Pan-Cancer Analysis of the Phosphorylation of HSF1

**Figure 6:**
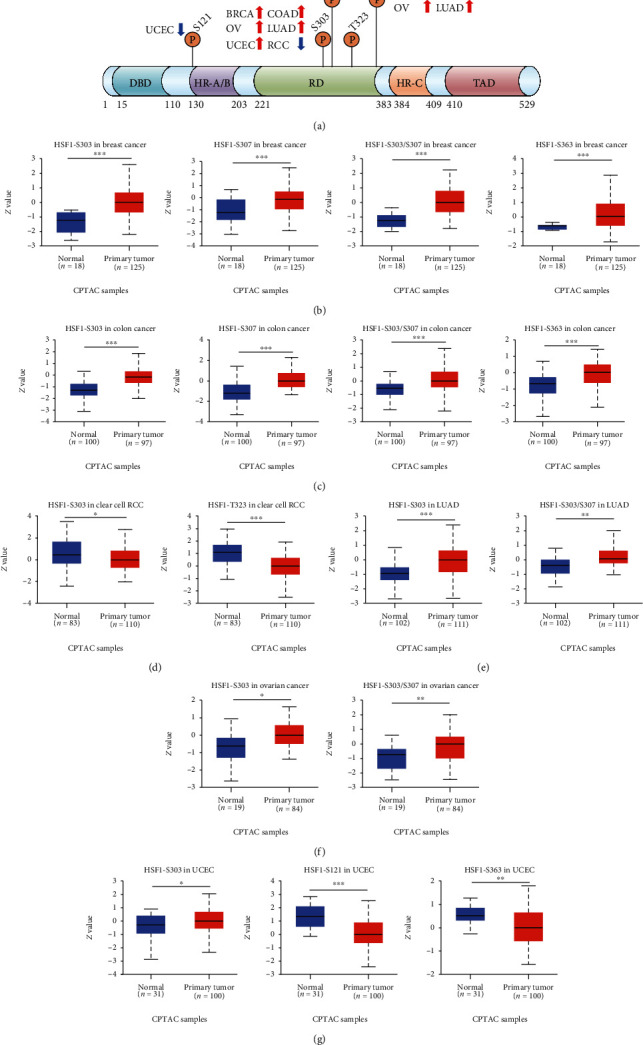
Phosphorylation of HSF1 in several selected cancers according to the CPTAC database. (a) The schematic diagram and phosphorylation sites of the HSF1 protein are shown. The phosphorylation of HSF1 at S303, S307, S303/S307, S363, S121, and T323 was analyzed in breast cancer (b), colon cancer (c), clear cell RCC (d), LUAD (e), ovarian cancer (f), and UCEC (g). The results were obtained from the UALCAN database. ^∗^*p* < 0.05, ^∗∗^*p* < 0.01, and ^∗∗∗^*p* < 0.001.

Posttranslational modification (PTM) is a key molecular mechanism of HSF1 activation [11–13]. Therefore, we examined alterations in HSF1 phosphorylation levels between primary tumor tissues and normal tissues. The CPTAC database includes six types of cancer, namely, breast cancer, clear cell RCC, colon cancer, LUAD, ovarian cancer, and UCEC (Figure 6(a)). Higher levels of S303 phosphorylation of HSF1 were observed in breast cancer, colon cancer, LUAD, ovarian cancer, and UCEC samples compared to normal samples (Figures 6(b), 6(c), and 6(e)–6(g). In contrast, S303 phosphorylation of HSF1 was decreased in clear cell RCC (Figure 6(d)). S307 phosphorylation of HSF1 was increased in breast cancer and colon cancer tissues compared to normal tissues (Figures 6(b) and 6(c)). S303 and S307 phosphorylation of HSF1 was significantly increased in breast cancer, colon cancer, LUAD, and ovarian cancer (Figures 6(b), 6(c), 6(e), and 6(f). S363 phosphorylation of HSF1 was remarkably increased in breast cancer and colon cancer, but decreased in UCEC tissues compared to normal adjacent tissues (Figures 6(b), 6(c), and 6(g)). Decreased T323 and S121 phosphorylation in HSF1 was observed in clear cell RCC and UCEC, respectively (Figures 6(d) and 6(g)). These findings suggest that the phosphorylation of the S303 and S307 residues of HSF1 plays a role in oncogenesis.

### 3.6. Functional Enrichment Analysis of HSF1 in COAD

**Figure 7:**
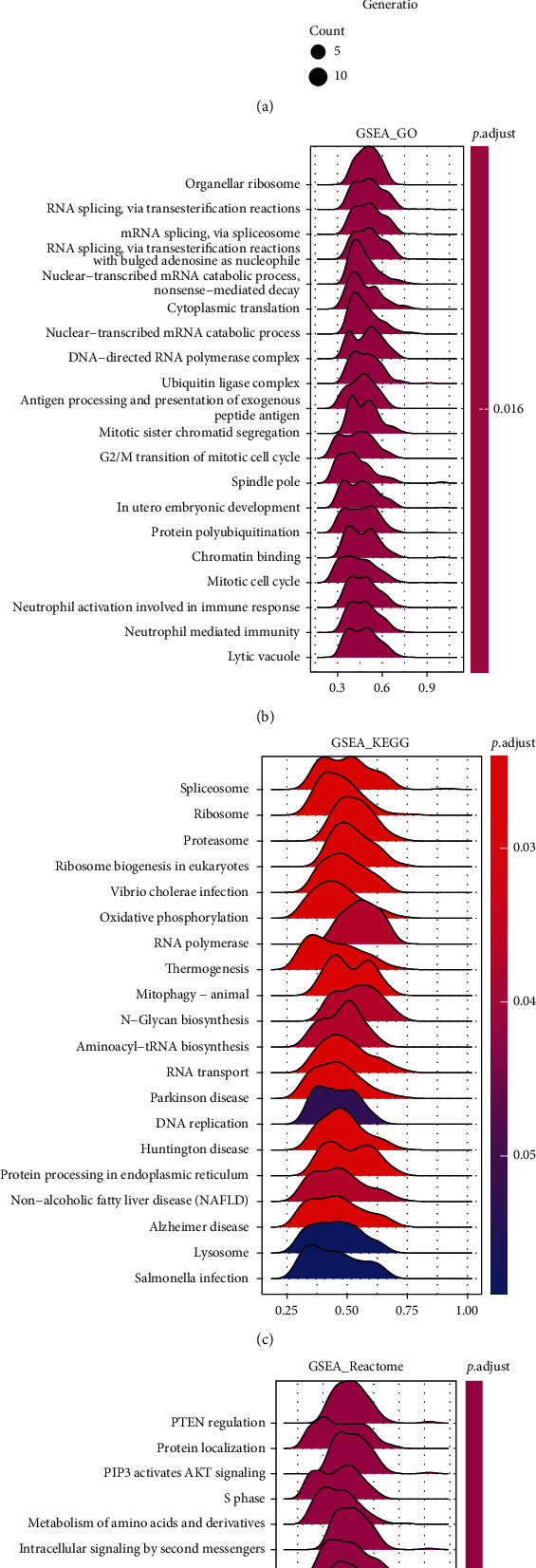
Merged enrichment plots for HSF1 obtained from KEGG and GSEA. (a) Top 20 pathways enriched in the KEGG analysis in COAD. (b)–(d) Merged plots of GSEA indicating the signaling pathways associated with HSF1 expression according to GO, KEGG, and Reactome analyses in COAD.

To deeply examine the molecular mechanisms of HSF1 regulation in diverse tumors, we performed Gene Set Enrichment Analysis (GSEA) and Kyoto Encyclopedia of Genes and Genomes (KEGG) analysis for COAD. The top 20 significant terms of the KEGG analysis included endocytosis, Alzheimer's disease, the spliceosome, the mTOR signaling pathway, RNA transport, insulin resistance, autophagy, and the Notch signaling pathway, and these pathways were associated with HSF1 (Figure 7(a)). Multiple bacterial or vital infection processes, including herpes simplex virus 1 infection, human papillomavirus infection, human immunodeficiency virus 1 infection, hepatitis B, and higellosis, also correlated with HSF1 (Figure 7(a)). Notably, we found that the HSF1 expression correlated with the PD-L1 expression and the PD-1 checkpoint pathway in COAD (Figure 7(a)).

GSEA was performed to examine HSF1-associated signaling pathways that were differentially activated in cancer. GSEA results revealed that HSF1 affected several GO terms, including the regulation of cell cycle phase transition, proteasomal protein catabolic process, mitotic nuclear division, membrane docking, chromatin remodeling, and histone demethylation (Figure 7(b)). GSEA results of KEGG analysis indicated that HSF1 was involved in various pathways, such as the spliceosome, ribosome biogenesis, Vibrio cholera infection, oxidative phosphorylation, mitophagy, thermogenesis, and neurodegenerative diseases (Figure 7(c)). The GSEA results for reactome terms suggested that several immune functional gene sets, including neutrophil degranulation, the adaptive immune system, and the innate immune system, were enriched in COAD (Figure 7(d)). These results suggest that HSF1 plays an important role in the inflammatory response and TME.

### 3.7. Pan-Cancer Analysis of the HSF1 Expression and Immune Cell Infiltration

**Figure 8:**
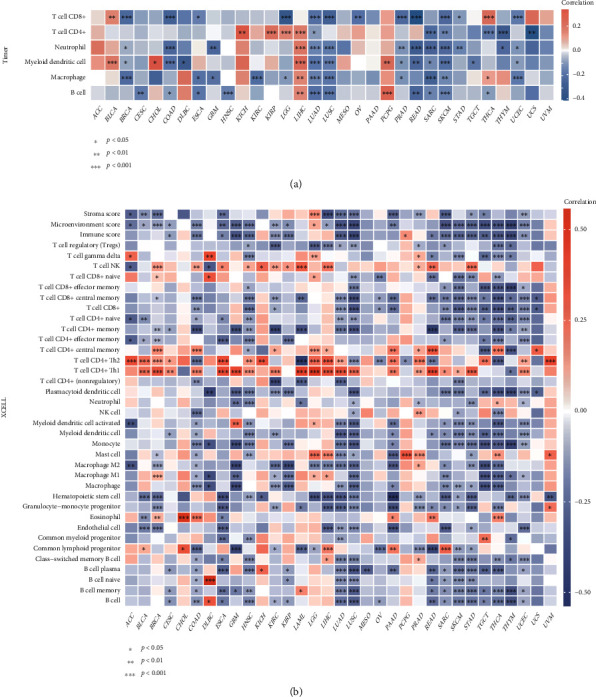
The HSF1 expression correlated with immune infiltration. (a) The HSF1 expression significantly correlated with the infiltration levels of various immune cells in the TIMER database. (b) The HSF1 expression significantly correlated with the infiltration levels of various immune cells based on xCell. ^∗^*p* < 0.05, ^∗∗^*p* < 0.01, and ^∗∗∗^*p* < 0.001.

Because of the distinct relationship between HSF1 and the immune response, we performed a pan-cancer analysis of the association between the HSF1 expression and the immune infiltration level based on the TIMER database. As shown in Figure 8(a), the expression of HSF1 was significantly associated with the abundance of infiltrating immune cells: B cells in 12 types of cancer, CD4+ T cells in 10 types of cancer, CD8+ T cells in 14 types of cancer, macrophages in 13 types of cancer, neutrophils in 13 types of cancer, and DCs in 15 types of cancer.

We further used the xCell online tool to examine the relationship between HSF1 expression and the infiltration of different types of immune cell subtypes. Among 38 subtypes of immune cells, we found that the HSF1 expression negatively and significantly correlated with these subtypes in LUAD, LUSC, SARC, SKCM, STAD, THCA, and UCEC (Figure 8(b)). Th1 and Th2 CD4+ T cells were most positively associated with the HSF1 expression in these different cancers (Figure 8(b)).

### 3.8. Pan-Cancer Analysis of the Correlation between the HSF1 Expression and Immune Modulators, TMB, and MSI

**Figure 9:**
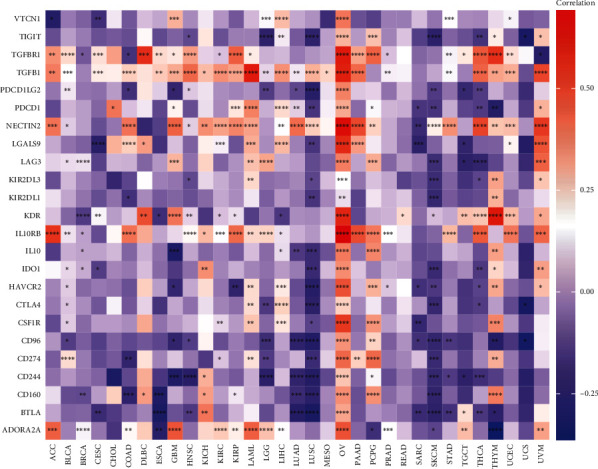
Correlation analyses of the HSF1 expression with immune checkpoint genes in pan-cancer. ^∗^*p* < 0.05, ^∗∗^*p* < 0.01, ^∗∗∗^*p* < 0.001, and ^∗∗∗∗^*p* < 0.0001.

Immunosurveillance influences the prognosis of cancer patients, and tumors evade immune responses by taking advantage of immune checkpoints, such as PD-1, PD-L1, and CTLA-4 [21, 22]. To closely estimate the association between the HSF1 expression and the TME in a pan-cancer dataset, we further investigated the relationships between the HSF1 expression and two major types of immune modulators. Notably, we observed that the expression of HSF1 negatively correlated with most immunoinhibitors and immunostimulators in LUAD, LUSC, and SKCM (Figure 9 and Supplementary Figure 5). In contrast, the expression of HSF1 positively correlated with most immunoinhibitors and immunostimulators in OV, PCPG, and THYM (Figure 9 and Supplementary Figure 5).

**Figure 10:**
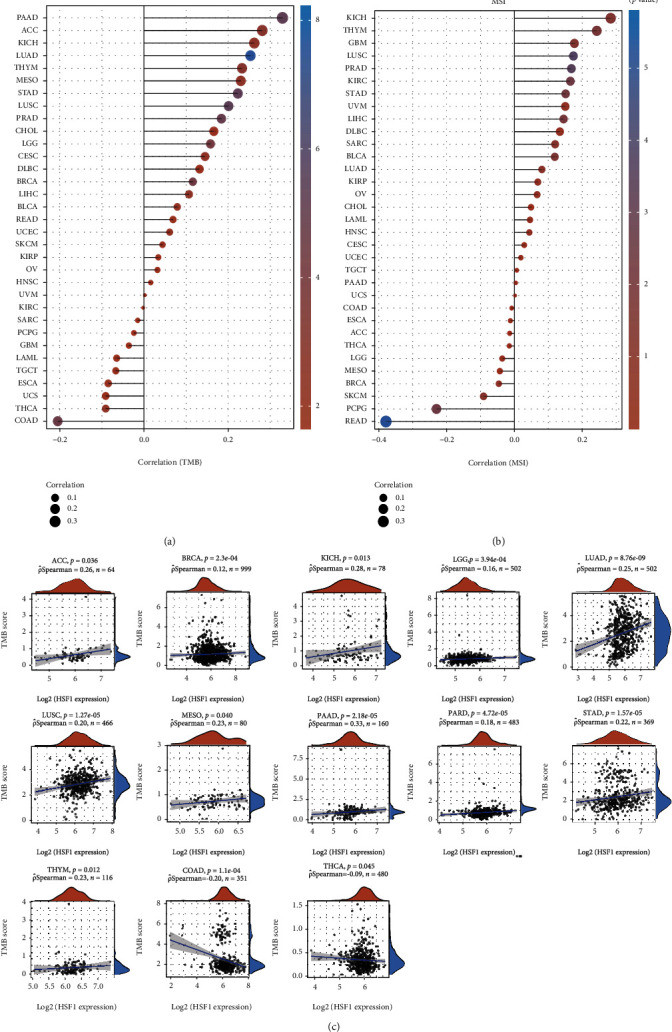
Correlation between the HSF1 gene expression and TMB and MSI in pan-cancer. (a) A stick chart shows the relationship between the HSF1 gene expression and TMB in diverse tumors. The red curve represents the correlation coefficient, and the blue value represents the range. (b) A stick chart shows the association between the HSF1 gene expression and MSI in diverse tumors. (d) Relationship between the HSF1 gene expression and TMB in pan-cancer. Correlation analysis was performed using Spearman's method.

TMB and MSI are two emerging biomarkers associated with the immunotherapy response. The relationship between the HSF1 expression and TMB was investigated. The expression level of HSF1 remarkably correlated with TMB in several tumors, including PAAD, LUAD, STAD, LUSC, PRAD, LGG, BRCA, and COAD (Figures 10(a) and 10(c)). The correlation of the HSF1 expression with MSI was also investigated in 33 types of cancer, LUSC, PRAD, KIRC and STAD exhibited positive correlations, and READ and PCPG exhibited negative correlations (Figure 10(b) and Supplementary Figure 6).

## 4. Discussion

Cancer is a serious threat to human health due to its high morbidity and mortality [1, 2]. The three most common cancers worldwide are breast, lung, and colon cancer [1]. The most common cancer treatments include surgical resection, radiation, and adjuvant chemotherapy, but their effectiveness remains limited [2]. Early detection and effective treatment are important prerequisites for improving the prognosis of cancer patients. Pan-cancer analysis could reveal the similarities and differences between different cancers and provide deep insights for the design of cancer prevention and personalized treatment strategies. A growing number of recent studies focused on genome-wide pan-cancer analysis to reveal gene mutations, RNA changes, and cancer-driving genes related to the initiation and development of cancer, which are of great significance for the early diagnosis of cancer and the identification of sensitive biomarkers [23, 24]. The present study first comprehensively examined the expression of HSF1 in a pan-cancer dataset. The results from the analysis of 33 cancer data sets from the TCGA were consistent with previous studies and demonstrated that HSF1 was significantly upregulated in BLCA, BRCA, CHOL, COAD, ESCA, GBM, HNSC, KICH, KIRC, LIHC, LUAD, LUSC, PRAD, READ, STAD, THCA, and UCEC compared to paracancerous and normal tissues (Figures 1(a) and 1(b)). The upregulated expression of HSF1 correlated with worse OS, DSS, DFI, or PFI in several cancers (Figures 2 and 3 and Supplementary Figures 1 and 2). The HSF1 expression was significantly associated with immune infiltration and immune checkpoint markers in various types of cancer (Figures 8 and 9). Genetic studies in cancer cells and normal cells demonstrated that HSF1 orchestrated the transcriptional regulation of genes aside from heat shock genes, which suggests that it participates in tumorigenesis by driving various unique signaling pathways and oncogenesis-related genes [15, 18]. Consistent with previous studies, our KEGG analysis suggested that HSF1 was significantly associated with many signaling pathways (Figure 7). Together, our study provides insights into the application of HSF1 as a potential prognostic biomarker in several cancers in the context of immunooncology and contributes to the development of HSF1-targeting therapeutic strategies.

HSF1 exerts a pleiotropic effect on malignancy because it may play roles in many aspects of tumor biology, including DNA repair, angiogenesis, and metabolism [8]. We and other researchers showed that HSF1 participated in oncogenesis by cooperating with p53, RPA, ATF1, and SSBP1 [25–30]. HSF1 exists in an inactive form under normal physiological conditions, and its activation is tightly regulated via PTM [8–10]. Several types of PTMs, such as phosphorylation, acetylation, and sumoylation, were identified in HSF1. The phosphorylation of HSF1 affects HSF1 dissociation, trimer formation, nuclear translocation, and DNA binding activity. Notably, S326 is a dominant target for HSF1 activation. HSF1 phosphorylation at S326 was closely associated with the maintenance of cancer stem-like cells and a high level of S326 phosphorylation in HSF1 correlated with worse prognosis than a low level in ovarian cancer [31]. Elevated S326 phosphorylation of HSF1 was also significantly associated with the progression, invasion, and prognosis of hepatocellular carcinoma [32]. AKT-regulated phosphorylation of HSF1 at S326 promoted proliferation, epithelial-mesenchymal transition (EMT), and cancer stem-like traits in gallbladder cancer [33]. The phosphorylation of AKT at S473 and the phosphorylation of HSF1 at S326 were highly related to a shortened time to metastasis in breast cancer [33]. PIM2-regulated phosphorylation of HSF1 at Thr120 facilitated breast cancer oncogenesis in vivo and in vitro [34]. We recently showed that cyclosporin A enhanced the sensitivity of cancer cells to hyperthermia and chemotherapy by promoting the phosphorylation of HSF1 at S303 and S307 and suppressing the expression of HSPs [35]. Notably, higher levels of phosphorylation of S303 and 307 of HSF1 were clearly observed in breast cancer, ovarian cancer, colon cancer, and LUAD in the present study (Figure 6). The levels of phosphorylation of S363 in HSF1 were significantly enhanced in breast cancer, ovarian cancer, and colon cancer compared to normal adjacent tissues (Figure 6). These findings suggest that HSF1 phosphorylation plays a role in tumorigenesis. Unfortunately, we could not investigate changes in the phosphorylation of S326 or other PTMs in HSF1. HSF1 is maintained in an inactive state via the constitutive phosphorylation at S303, S307, and S363 [11–13]. A more comprehensive understanding of the positive and negative regulation of HSF1 in PTMs may alter the response to therapies that lead to HSF1 activation and help improve the efficacy of HSF1-targeted treatments.

DNA methylation is a major form of epigenetic modification of DNA that regulates the gene expression without altering the sequence of DNA [20]. DNA methylation generally suppresses the gene expression by changing chromatin structure, DNA stability, and DNA conformation [36]. The links between DNA methylation and cancer were gradually discovered in recent decades. Hypermethylation within promoter regions often leads to the silencing or inactivation of tumor suppressor genes in cancerous cells [20, 36]. The present study showed that DNA methylation of HSF1 was downregulated in most common cancers, which is consistent with the upregulation of the HSF1 expression (Figure 5(a) and Supplementary Figure 4). The relationship between DNA methylation and HSF1 expression warrants more indepth study.

Recently, numerous studies from invertebrate and vertebrate genetic systems suggested that HSF1 was widely associated with immunity. HSF1 is essential for the optimal immune response against various pathogenic infections in both *C. elegans* and animal models [37–39]. HSF1 regulates the expression of inflammatory cytokines and cytokine receptors, such as interleukin 1 beta (IL-1b), IL-6, tumor necrosis factor- alpha (TNF-*α*, granulocyte colony stimulating factor (G-CSF), and macrophage colony-stimulating factor (M-SCF) [40–44]. HSF1 suppresses the expression of these inflammatory genes via direct binding to the promoter region of target genes or facilitation of the binding of other transcription factors that negatively regulate the gene expression [42, 43]. In contrast, HSF1 indirectly mediated the IL-6 expression via activating transcription factor (ATF3) in lipopolysaccharide- (LPS-) induced liver injury [40]. The silencing of HSF1 suppressed the activation of Snail and increased the activation of the innate immune signaling receptor NLRP3 in a mouse model of liver ischemia-reperfusion (IR) injury. In contrast, the adoptive transfer of HSF1-expressing macrophages to myeloid-specific Notch1 knockout mice promoted Snail activation and alleviated IR-triggered liver inflammation, which suggests that the Notch1/HSF1/Snail axis is a therapeutic target for liver inflammatory injury [45]. Hypercapnia increased the HSF1 expression and nuclear translocation to promote its activation in an alveolar macrophage cell line and primary murine alveolar macrophages [46]. Knockdown of HSF1 increased LPS-induced IL-6 and TNF-*α* release, likely via negative regulation of NF-*κ*B activity [46]. IgG generation was also impaired in HSF1-/- mice [47]. Deletion of HSF1 in mice aggravated lung damage and macrophage infiltration in LPS-induced acute lung injury. HSF1 suppressed the transcription of MCP-1 and its receptor CCR2 via direct binding to heat shock elements (HSEs) in the promoters of these inflammatory genes to inhibit macrophage infiltration [48]. HSF1 is also deeply involved in the inflammatory response during HIV infection via competition with NF-*κ*B in the nucleus [49]. Consistent with previous studies, our GO analysis revealed that inflammatory-associated pathways, such as neutrophil activation in the immune response and neutrophil-mediated immunity, were closely associated with HSF1 (Figure 7). The Reactome analysis showed that HSF1 significantly correlated with neutrophil degranulation, the adaptive immune system, and the innate immune system (Figure 7). These findings suggest that HSF1 plays a complex role in regulating immunity.

The TME is a complex structure composed of tumor cells, nonmalignant cells, blood vessels, extracellular matrix, and other substances [21, 22]. Different types of immune cells play key regulatory roles in the TME. Increasing evidence suggests that the interaction between cancer cells and various components of the TME facilitates immune escape of tumors and ultimately results in tumor proliferation, recurrence, and metastasis. Although immunotherapy made some breakthroughs in cancer treatment, there are many challenges in its successful application [50, 51]. Therefore, the identification of new targets and biomarkers is key to further improving the efficacy of immunotherapy. A comprehensive understanding of the status of immune infiltration in cancer patients is particularly important for selecting the correct individualized immunotherapy strategy [50, 51]. The function of HSF1 and its impact on the tumor immune microenvironment were not fully investigated. The present study revealed the relationship between HSF1 and tumor immune cells and investigated the immune status of cancer patients by examining the HSF1 expression. We found that HSF1 significantly correlated with the infiltration levels of B cells, CD8+ T cells, CD4+ T cells, macrophages, neutrophils, and DCs in many cancers (Figure 8). The relationships between the HSF1 expression and immunosuppressive and immunostimulatory genes were also analyzed. There was a high correlation of the HSF1 expression with most immunosuppressive and immunostimulatory molecules in LUAD, LUSC, SKCM, OV, PCPG, and THYM (Figure 9 and Supplementary Figure 5). Notably, our findings revealed an interesting phenomenon in which most immunosuppressive and immunostimulatory molecules negatively correlated with the HSF1 expression (Figure 9 and Supplementary Figure 5). The causes of primary and secondary immunotherapy resistance are multifaceted and include internal factors of the tumor and the complex interaction between cancer cells and various components of the TME. The negative correlation between immunosuppressants and immunostimulants in the same group of patients further reflects the complexity of the TME.

## 5. Conclusions

The upregulation of the HSF1 expression corresponded to a poor prognosis in patients and correlated with the infiltration levels of B cells, CD8+ T cells, CD4+ T cells, macrophages, neutrophils, and DCs in diverse cancers. Increased HSF1 phosphorylation and decreased HSF1 methylation were observed in many types of cancer. The HSF1 expression was significantly associated with the expression of immune checkpoint markers. Future prospective and experimental studies of the HSF1 expression and immune cell infiltration in different cancer populations may provide additional insights into the tumor mechanisms and the development of therapeutic strategies targeting HSF1 to improve the therapeutic efficacy of immunotherapy.

## Data Availability

The data used to support the findings of this study are available from the corresponding author upon request.

## Supplementary Materials

Supplementary Materials Supplementary Figure 1: association between the HSF1 expression and PFI of cancer patients. (a) A forest plot of hazard ratios of HSF1 in 32 types of tumors. (b) Kaplan-Meier survival curves of PFI for patients stratified by the different expressions of HSF1 in ACC, PRAD, HNSC, and UCS. Supplementary Figure 2: association between the HSF1 expression and DFI in cancer patients. (a) A forest plot of hazard ratios of HSF1 in 28 types of tumors. (b) Kaplan-Meier survival curves of DFI for patients stratified by the different expressions of HSF1 in ACC and TGCT. Supplementary Figure 3: promoter methylation level of HSF1 in pan-cancer. The results were obtained from the UALCAN database. ^∗^*p* < 0.05, ^∗∗^*p* < 0.01, ^∗∗∗^*p* < 0.001, and ^∗∗∗∗^*p* < 0.0001. Supplementary Figure 4: the correlation between the HSF1 expression and the methylation level of HSF1 in pan-cancer. ^∗^*p* < 0.05, ^∗∗^*p* < 0.01, and ^∗∗∗^*p* < 0.001. Supplementary Figure 5: correlation analysis of the HSF1 expression with immunostimulatory genes in pan-cancer. ^∗^*p* < 0.05, ^∗∗^*p* < 0.01, ^∗∗∗^*p* < 0.001, and ^∗∗∗∗^*p* < 0.0001. Supplementary Figure 6: relationship between the HSF1 gene expression and MSI in pan-cancer. Correlation analysis was performed using Spearman's method.
